# Situational analysis of antimicrobial stewardship program (ASP) among public and private sector tertiary care hospitals in Karachi, Pakistan: A qualitative case study

**DOI:** 10.1017/ash.2023.427

**Published:** 2023-09-22

**Authors:** Asma Pethani, Atif Riaz, Shagufta Perveen, Ali Faisal Saleem

**Affiliations:** 1 Department of Paediatrics and Child Health, The Aga Khan University, Karachi, Pakistan; 2 Department of Community Health Sciences, The Aga Khan University, Karachi, Pakistan

**Keywords:** Antimicrobial resistance, Antimicrobial stewardship, Implementation, Pakistan, Qualitative

## Abstract

**Objective:**

To assess the current status of implementation of the Antimicrobial Stewardship Program (ASP) across Tertiary Care Hospitals in Karachi, Pakistan.

**Design:**

Exploratory qualitative case study.

**Setting:**

Public and private tertiary care hospitals in Karachi, Pakistan

**Participants and Methods:**

The study data were collected from 3 public and 4 private tertiary care hospitals. Twenty-eight in-depth interviews were conducted from the Chief Executive Officer, Chief Medical Officer, Medical Superintendent, and departmental heads of internal medicine, general surgery, and pediatric, respectively. Purposive sampling was done to include higher and middle managers, whereas the infectious diseases consultant, infectious diseases/clinical pharmacist, and clinical microbiologist were interviewed through snowball sampling methodology. Analysis was done using NVivo. Data were source-triangulated within and among the study setting and study participants.

**Results:**

We found that more than two-thirds (n = 5, 71%) of tertiary care hospitals in Karachi do not have a structured ASP which includes major public sector hospitals (n = 3, 43%) and half of the private sector hospitals (n = 4, 29%). The study results led to four broad themes, (1) ASP structure, (2) ASP interventions, (3) hospital medical record-keeping system, and (4) structured way for analyzing and reporting mechanism of data related to the ASP. At H1 and H2, there was a consistency in ASP structure and interventions, whereas paucity seen among remaining tertiary care hospitals.

**Conclusion:**

There is an alarming need for ASP in the public and private sector hospitals in Karachi. This study can inform future stakeholders regarding ASP and strategies for structural change at hospitals.

## Introduction

Antimicrobial resistance is a substantial threat to global health.^1^ Antimicrobial resistance refers to the change in ways microorganisms render antimicrobial drugs ineffective.^2^ It is promulgated by misuse of antimicrobial medicines, non-existent or inadequate infection control programs, weak laboratory capacities, poor quality or counterfeit drugs, ineffective surveillance, and regulations for the use of antimicrobial medicines.^3^ Healthcare professionals should be committed to the responsible use of antibiotics. The stewardship programs not only improve outcomes but also save pharmacy costs in health care.^3^

In Pakistan, the average number of drugs prescribed per patient is 3 or more in as compared to an average of 2-3 in LMICs.^4–6^ ASP has improved antimicrobial prescriptions, and its utilization has shown decreased rates of AMR infections in hospitals.^7–9^ In most of the developing countries including Pakistan, little effort has been made despite the high prevalence of infectious diseases. A gap exists regarding knowledge of healthcare personals on ASP due to lack of surveillance at the provincial and national level.^4^

In March 2014, the Medical Microbiology and Infectious Diseases Society of Pakistan (MMIDSP) launched an Antibiotic Stewardship Initiative in Pakistan,^10^ with the involvement of major stakeholders of both public and private institutions. However, there exists scarcity of available literature about ASP development and implementation in Pakistan. This study aimed to assess the status of implementation of the ASP across Karachi, Pakistan.

## Methods

### Study design

An exploratory case study was conducted to gain knowledge about the development and implementation of the ASP at the organizational level in Karachi, Pakistan. Based on Yin’s definition of the case study approach,^11^ we have investigated the phenomenon of ASP which served as the “case” with the context of public and private tertiary care hospitals to understand and assess the implementation of ASP. This research study is aligned with the reporting recommendations from COREQ-32 criteria.^12^

### Study setting

The study was conducted in Karachi, which is the largest city and financial hub of Pakistan. At the time of the study, there were 3 public and 7 private tertiary care hospitals in Karachi, Pakistan.

### Recruitment and sampling

**Figure 1.**
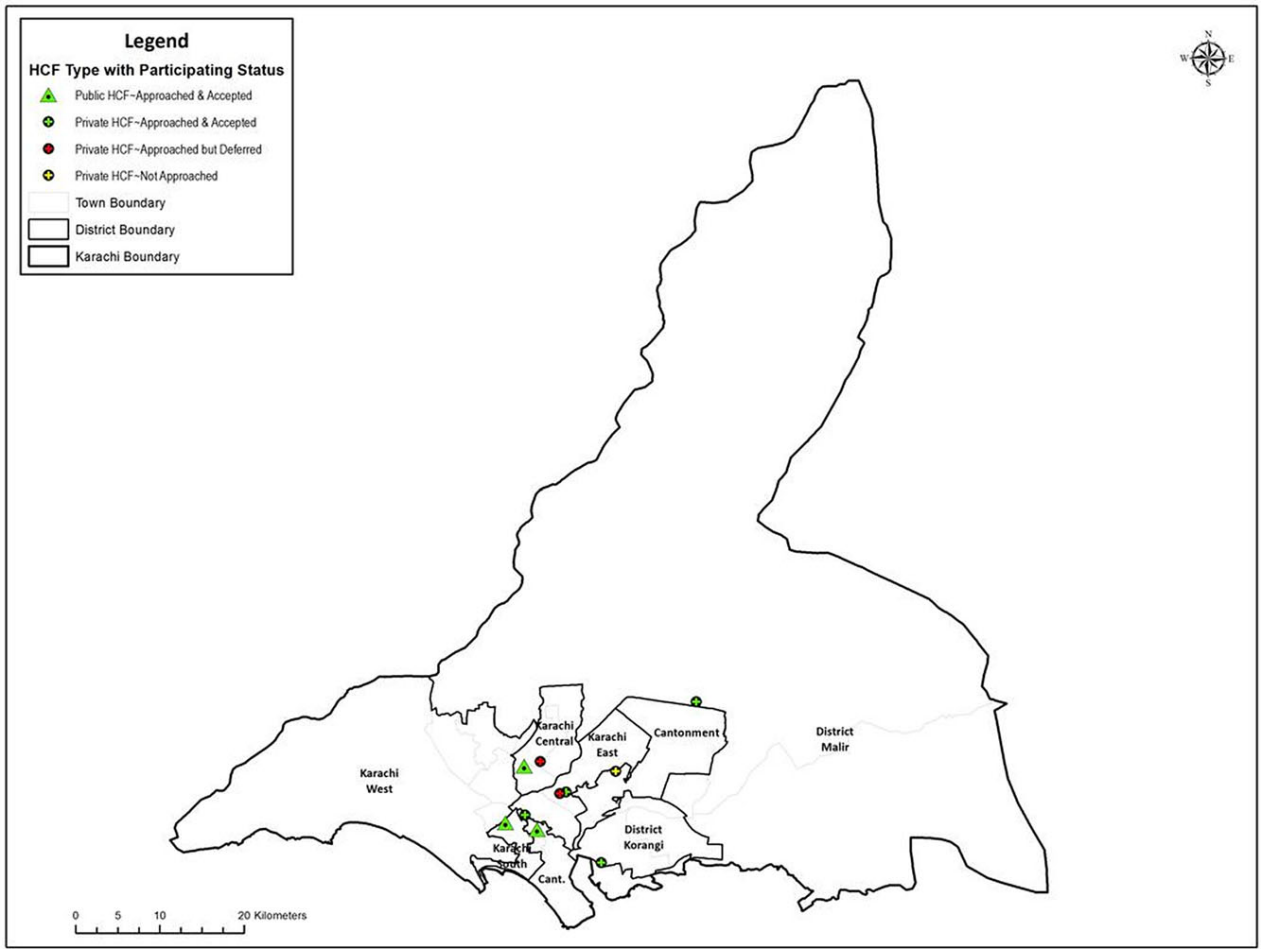
Participating tertiary care hospitals

All the tertiary care hospitals were approached, of which 3 public and 4 private tertiary care hospitals consented to participate in the study. The study participants included higher management (Medical Superintendent/Medical Director) and middle management (Departmental Heads) responsible for ASP development and implementation at public and private tertiary healthcare hospitals. The selected participating tertiary care hospitals are given in Figure 1.

### Pilot testing and data collection

An open-ended, semi-structured questionnaire for an in-depth interview was used as a data collection tool. The interview guide was prepared with the help of the CDC assessment tool and global literature. The questionnaire was pre-tested on two individuals randomly selected from participating institutes. Probes, improper sequencing, and question phrasing were amended after the pilot testing. A free flow of information was encouraged, using probes from arising interview. A note-taker was designated to document all notes during interviews. All interviews were audio-recorded after taking consent from the participants and transcribed verbatim which ranged between 30 and 60 min.

### Ethical approval

Ethical clearance was obtained from the Ethical Review Committee [ERC] of the Aga Khan University and Institutional Review Boards [IRB] of all participating hospitals.

### Informed and voluntary consent

The research team obtained informed written consent which explained the purpose of this study, its risks, benefits, and respondent rights. The participants were encouraged to ask questions and were informed that their identities will not be disclosed to anyone. There was no utilization of coercive techniques for recruiting participants.

### Confidentiality

In-depth interviews were conducted in a separate room under strict privacy measures. The data from study participants were kept confidential under the lock and key. Pseudonyms were used to keep the participant’s identity confidential. The data were utilized only for study purposes and access to information was limited to the study team only.

## Analysis

Data were analyzed with the assistance of NVivo 12 software (QSR International). The interviews were transcribed and kept in a password-protected secured file. Coding of data into nodes, sub-nodes was done. Similar nodes were categorized into themes and sub-themes and brought together in the result section. The emphasis was on the formative underlying meaning of the transcripts thus providing an account of the status of ASP implementation among public and private tertiary healthcare institutes. Data for each code were repeatedly reviewed to ensure conceptual consistency until a final coding was produced that covered all relevant themes identified in the text.

## Results

**Figure 2.**
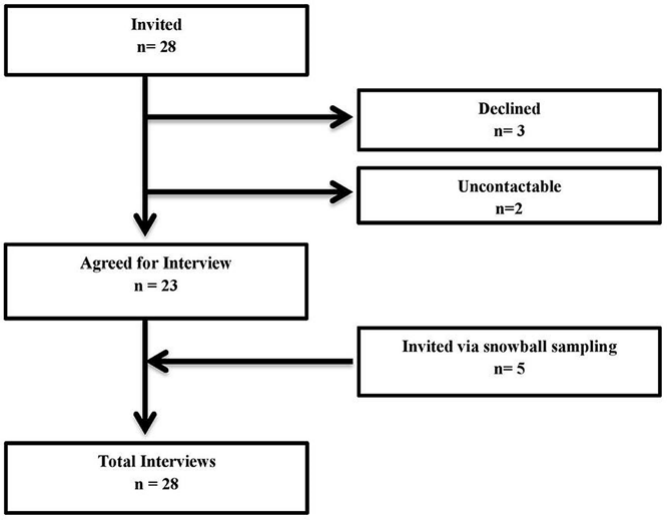
Flow of participants through the study

Twenty-eight in-depth interviews were conducted. Figure 2 represents the flow of participants through the study. Out of 28 respondents, 5 were from the higher executive, 18 were clinical departmental heads and associate professors, 2 from the Department of Pharmacy, 1 was infection control head, and 1 was clinical microbiologist (Table 1). It was observed that ASP is implemented at two private tertiary care hospitals; however; the Department of Medicine at a public sector tertiary hospital had initiated it. The remaining five tertiary care hospitals which included Private (n = 2) and Public (n = 3) had not initiated an ASP till then. Particulars regarding hospital characteristics and their ASP details are provided in Tables 2 and 3, respectively. Results are presented under 4 main themes: ASP Structure, ASP interventions, Medical Record-Keeping System, and Analyzing and Reporting mechanism of ASP. *Supplementary Data—Examples of initial coding, sub-themes, and themes* provides an example of how the analysis was done from initial coding to the final themes and sub-themes. Quotes are presented to support analysis and are labeled by the participant and hospital number.

**Table 1.** Demographics of qualitative participants

Participants designation	Participants number	Qualification	Years of experience
CMO/Medical Director/ Medical Superintendent	5	MBBS, FCPS, Public Health	21-30 yr
Departmental Heads (Medicine, Surgery and Pediatrics)	18	MBBS, FCPS, Diplomate of American Board	16-35 yr
ASP—Chair	1	MBBS, ABIM	>30 yr
ID/Clinical Pharmacist	2	Pharm D	6-18 yr
Infection Control Nurse	1	MScN	20 yr
Clinical Microbiologist	1	MSc Medical Technology	19 yr

**Table 2.** Hospital characteristics

Hospital	H1	H2	H3	H4	H5	H6	H7
**Type**	Academic/Tertiary Care	Tertiary Care	Academic/Tertiary Care	Academic/Tertiary Care	Academic/Tertiary Care	Academic/Tertiary Care	Academic/Tertiary Care
**No. of beds**	716	200 +	1800 +	500	–	250	1500
**Sector**	Private	Private	Public	Private	Public	Private	Public

**Table 3.** Antimicrobial stewardship program details

ASP organization				
	H1	H2	H3	
**Existence of ASP (Current form)**	January 2018	10 years	Not existed institutionally but implemented by one of the department	
**Implementation status**	Complete implementation	Partial implementation	Institutional	Departmental (50 Beds)
			Not implemented	Partial implementation
**Stewards**	-ASP chair -Pharmacy and therapeutic committee -ID consultant -ID pharmacist -Infection control nurse -Microbiologist	-ASP chair -Pharmacy department -ID consultant -Infection control Nurse -Microbiologist		Partial implementation -Head of the department -Senior consultant -Clinical pharmacist (Not trained in infectious diseases)
**Dedicated ASP time (full-time)**	No	No	–	No
**Microbiology Involved**	Yes	Yes		Not directly involved
**Pharmacy Involved**	Yes	Yes to an extent		Departmental initiative
**ASP measures and interventions**				
** Dedicated ASP phone number or direct line**	Yes	Yes		Departmental steward’s direct number
** Mode of consultation**	-Bedside consultations -Phone	-Bedside consultations -Phone		-Bedside consultation
** Systematic counseling**	-Laboratory results -Pharmacy alerts	-Laboratory results		-Microbiology lab results
** Infectious Diseases Consultant onwards**	No solicited consultation	No solicited consultation		-Routine ward rounds by departmental stewards -In case of complex cases takes help from ID consultant available at associated institute.

## Theme 1: ASP structure

### ASP implementers

- ***Designated committee:***

Both hospitals *H1* and *H2* had a method of the designated multidisciplinary committee to oversee and coordinate ASP. The committee was chaired by pediatrics and adult infectious diseases consultants at *H1* and *H2,* respectively. Both hospital committees involved hospital administration, infection prevention and control professionals, physicians, microbiologist, nurses, information technologist, and epidemiologist except for infectious diseases pharmacist who is only involved at *H1*. *(Refer to supplementary data; category—1.1.1)*.

1. ***Role and responsibilities of stewards:***

The participant (01) at *H1* mentioned that ASP chair was responsible for clinical guidelines to support stewards. The ID pharmacist was responsible for antimicrobial reviews according to susceptibility data provided by the microbiology laboratory. *(Refer to supplementary data; category—1.1.2)*.

### Leadership commitment for ASP

- ***Hospital leadership support:***

Extraordinary support by the hospital leadership is the formal approval to bring about day-to-day activities of the stewardship program, stated by interviewees. At *H1,* the pilot project was initiated at the surgical ICU for stewardship interventions under the supervision of hospital leadership and departmental head. When the pilot project became successful and showed constructive results, the ASP was formally instituted with an official statement by the hospital’s higher management. However, one of the key respondents from *H2* stated that there is a lack of leadership commitment for the implementation of the antimicrobial stewardship program (Refer to supplementary data; category—1.3.1).

1. ***Hospital leadership commitment for allocation of resources:***

The hospital leadership at *H1* and *H2* demonstrated willingness for the allocation of finances, human resources, information technology, and education to the stewards as a commitment toward antimicrobial stewardship activities (Refer to supplementary data; category—1.3.2).

## Theme 2: ASP interventions

### Formulary restrictions and preauthorization:

The participants from *H1* described the formulary restriction and preauthorization as one of the core intervention for ASP. It involved restricting the use of certain antimicrobials to specific indications and duration of therapy. (Refer to supplementary data; category 2.1.1).

- ***Documented policy for restricted and controlled antimicrobials:***

The responder from *H1* stated that the institute’s official documented policy regarding the restricted and controlled antimicrobial policy is available. The policy is to delineate procedures for judicious use of restricted and controlled antimicrobials. However, at *H2* and *H3* the participants stated that no such policies exist. At *H2,* any consultant physician can prescribe broad-spectrum antimicrobials without consultation or approval from the infectious diseases consultant. However, the key stakeholder of the stewardship committee stated that there has been a discussion regarding the sanctioning of preauthorization strategy to restrict the use of certain antimicrobials but still lacks consensus. The departmental initiative at *H3* was restricting the prescription of broad-spectrum antimicrobials by restricting its availability at the pharmacy. (Refer to supplementary data; category—2.1.2).

1. ***Documented restricted and controlled antimicrobials/ Antimicrobial drugs and regimens require ID approval before prescribing:***

The respondents from the private tertiary care hospital *H1* specified the institutional document restricted and controlled antimicrobials and regimens which require prior approval for prescribing. The documented restricted and controlled antimicrobials include fosfomycin IV, tigecycline, linezolid IV/PO, caspofungin, trimethoprim/sulfamethoxazoles IV, carbapenems, antiretroviral medicines, and colistin. These drugs needed ID approval before prescription (Refer to supplementary data; category—2.1.3).

1. ***General prescribing policy for restricted and controlled antimicrobials in the life-threatening situation:***

The participants (*H1*) stated empirically single dose could be prescribed in case of life-threatening situations of the restricted and controlled antimicrobials because they did not want to delay the care for that particular patient. However, subsequent doses have to be approved by the ID pharmacist/ ID physician before they can be provided (Refer to supplementary data; category—2.1.4).

1. ***Approval process for the restricted and control antimicrobials:***

The participants from *H1* and *H3* told about an approval process/system for restricted and controlled antimicrobials for which the formulary restrictions are obligatory. In the case of *H1* and *H3*, the ID consultant or ID pharmacist and head of department or clinical pharmacist are responsible for the approval, respectively (Refer to supplementary data; category—2.1.5).

### Prospective audit and feedback

The participants from *H1*, *H2,* and *H3* reported prospective audits and feedback as the core intervention of stewardship activities. The approach aims to suggest changes and provide patient-specific education for improving antimicrobial utilization, optimizing, and streamlining therapy. When suitable, recommendations are sometimes made to intensify treatment to increase therapeutic efficacy. The participants reported the antimicrobials or combinations which need to be audited are Vancomycin + Meropenem for >3 days, IV Colistin for > 3 days, Tazobactam and Meropenem (Refer to supplementary data; sub-theme—2.2).

### Supplemental elements of antimicrobial stewardship program

- ***Parenteral to the oral conversion of antimicrobials:***

The institutes conversed regarding the targeted strategies for rationalizing antimicrobials. The criterion for the intervention is such that the review of antimicrobials takes place after 48 hours of initiating the therapy, if the patient has a functioning gastrointestinal tract and is clinically stable, that is afebrile (temperature <38°C or 100.4°F) for at least 24 hours and WBC counts are normalizing (Refer to supplementary data; category—2.3.1).

1. ***Dose adjustments and dose optimization:***

The second intervention that is conducted by the pharmacy department is dose adjustments and dose optimizations, based on the laboratory parameters like renal function tests, drug levels, site of infection, causative organism, and the pharmacokinetic and pharmacodynamics of antimicrobial agents. A pharmacist consult services are initiated at *H1* to support clinicians in optimizing the dose and frequency for individual patients (Refer to supplementary data; category—2.3.2).

1. ***Time-sensitive automatic stop orders:***

According to the participants, automatic stop orders decreased the excessive use of antibiotics for both prophylaxis and treatment. In the institutional automatic stop orders at *H1* and *H2*, the use of antimicrobials was legitimate for a defined period, after which the provider acquired approval from the designated steward for continuation. If approval wasn’t sought, the antimicrobial was automatically withdrawn (Refer to supplementary data; category—2.3.4).

## Theme 3: Medical record-keeping system

- ***Documentation policy for antimicrobial prescriptions:***

The participants emphasized the documentation policy for all the prescribed drugs. It is essential to document dose, duration, route, and indication to ensure that the selected antimicrobial agent is appropriate and rationalized. Both *H1* and *H2* had computerized physician order entry systems and electronic medical records. At *H3,* the computerized order entry and documentation facility is unavailable therefore the prescribers documented in patients’ record files (Refer to supplementary data; category 3.1).

1. ***Monitoring of documentation policy:***

The participants from ASP-implemented institutes reported that the ID pharmacists/Clinical pharmacist and departmental heads were responsible for monitoring documentation related to antimicrobial agents. The monitoring mechanism varied from a hospital to another. Generally, the monitoring was set up via the pharmacy login entries and daily clinical rounds. (Refer to supplementary data; category 3.3).

## Theme 4: Analyzing and reporting data of antimicrobial stewardship program

### Quality measures

According to all the participants, ASP comes up with the regulatory domain and measurement of antimicrobial prescribing. The reported quality measures were divided among the process and outcome measures Table 4). All these measured indicators were reported by H1 whereas H2 produced cumulative antibiogram and drug cost.

**Table 4.** Quality measures

**a. Process measure**	Patient management	✓ Antimicrobial de-escalation ✓ IV to PO conversion ✓ Double anaerobic therapy ✓ Infectious disease consultation performed
	Stewardship program	✓ Antimicrobial courses reviewed by ASP ✓ Compliance rate
	Consumption	✓ Defined daily doses per 1,000 patient days
**b. Outcome Measure**	Clinical	✓ Hospital-acquired infection rates ✓ Length of stay ✓ Mortality ✓ Antimicrobial resistance rate (cumulative antibiograms)
	Financial	✓ Drug cost

## Discussion

### Principal findings

The study highlights that there is a serious dearth of ASP in a major city of Pakistan. We found that five out of seven tertiary care hospitals lacked structured ASP which included hospitals from private and public sectors even though the Antimicrobial Resistance National Action Plan of Pakistan by the Ministry of National Health Sciences in May 2017 mentioned combatting AMR as a priority.^13^

The existence of ASP is regarded as chief quality improvement projects only at the two private tertiary care hospitals (*H1 and H2*), whereas at public tertiary care hospital (*H3*), only one of the departments had taken an initiative to implement it. The SHEA/IDSA recommends that an infectious disease physician and infectious disease pharmacist should lead the ASP team as they bring a different set of skills.^14^ This recommendation is in place at ASP-implemented institutions whereas the remaining must aim to institutionalize ASP.^15^ The principal responsibilities of ASP team members are to determine ASP goals, assisting and educating physicians and nurses about improving antimicrobial use, and resolving disparities among the ASP team and prescribers. The pharmacist is vital for ASP as they are experts in the dosing and monitoring of therapeutic drugs.^16,17^ This exemplary hospital leadership commitment was evident at *H1* and *H2*. The policy document, sustainable finances, human resource, and educational training support is the prime responsibility of hospital administration.^18^ These fundamental affirmances were seen at *H1* and *H2* only. The ASP committee is liable to report to the institutional leadership regarding stewardship data such as antimicrobial usage, compliance, and review of ASP strategies and issues faced by stewards not only for better accountability and clarity.^15^ It has been observed that the hospital (*H1*) has shown worthy leadership commitment whereas most tertiary care hospitals of Karachi are in dire need of such executive commitment.

At *H1,* the interventions are classified as core and supplemental interventions. In core interventions, formulary restrictions and prior authorization, prospective audit and feedback and antimicrobial clinical guidelines whereas in supplemental interventions pharmacy-driven strategies are structured. *H2* is implementing antimicrobial guidelines and prospective audit and feedback strategies. *H3* is implementing clinical guidelines, prior authorization, and prospective audit and feedback strategies. The most commonly used strategy under ASP is prospective audit and feedback in which interaction between the ASP team and prescribers is conducted for recommending a patient’s specific therapy.^19^ It is usually performed at 48 to 72 hours after the microbiology data are available and the patient’s clinical progression is further clarified. Different methods have been used to provide audit feedback; however, in the conducted study most common audit method was a face-to-face conversation and writing notes in the patient’s file.

The low-hanging fruit for ASP is pharmacy-driven interventions. These interventions are almost in place at all the ASP-implemented tertiary care hospitals. Literature has supported that by converting antimicrobials from IV to PO, ASP has demonstrated incredible cost savings without compromising patient’s safety and treatment effectiveness.^20^ The time-sensitive automatic stop order strategy was structured with the help of information technology at *H1* and *H2*. It is one of the effective ASP interventions which decreased unnecessary prescription of both treatment and prophylaxis antimicrobials.^21^ This intervention helps in encouraging prescribers for a re-evaluation of antimicrobials based on patient’s clinical symptoms and microbiological report to promote rational and safe use.^22^ Apart from pharmacy-driven interventions, education of prescribers and patients is been considered an effective strategy. *H1, H2, and H3* were carrying out mandatory sessions for the prescribers, interns, and students for rationalizing the use of antimicrobial agents while patients were provided with individual education as per their disease conditions.

The key component for the successful ASP is the measuring effectiveness of interventions. This includes total antimicrobials used, monitoring of structural and process indicators, and auditing quality of prescriptions. The indicated measurements all were reported by *H1,* whereas the *H2* was producing cumulative antibiogram and drug cost. However, the departmental initiative at *H3* had managed to get the ward-specific susceptibility data from the microbiology laboratory.

### Strengths and limitations

This is the first qualitative study about ASP development and implementation situational analysis at the tertiary care hospitals ever performed in Pakistan. It has attempted to include the maximum contribution of all major stakeholders involved from public and private tertiary care hospitals. The use of the triangulation technique by sources has increased the credibility of research by reducing the effect of the researcher’s biases. There are several limitations to the methods, that the study was only limited to Karachi due to the time constraint. However, the city has its unique geography, population, and health system. Hence, the results cannot be generalized to the rest of the country. It can be conducted in other provinces, for a better understanding because the irrational and non-judicious use of antimicrobials are common across the country, and there exists a lack of unified and standardized ASP.

## Conclusion

From the study, it is evident that public and private tertiary care hospitals within Karachi fall short in providing robust antimicrobial stewardship program. It is essential that hospitals implement an effective Antimicrobial Stewardship Program to ensure proper use of antimicrobials. It is important to have strong leadership commitment and their support toward adequate resources. Misuse of antimicrobials has resulted in increased resistance; therefore, there is a strong need for an appropriate mechanism at institutions to implement ASP.

## Supporting information

Pethani et al. supplementary material Pethani et al. supplementary material

## Supplementary material

For supplementary material accompanying this paper visit http://doi.org/10.1017/ash.2023.427. click here to view supplementary material

## Data sharing

All data relevant to the study are included in the article or uploaded as supplementary information.
