# Supplementary material for: Situational analysis of antimicrobial stewardship program (ASP) among public and private sector tertiary care hospitals in Karachi, Pakistan: A qualitative case study
Source: Antimicrob Steward Healthc Epidemiol. 2023 Sep 22;3(1):e161. doi: 10.1017/ash.2023.427 (PMC10523531; doi:10.1017/ash.2023.427)
Supplement: Pethani et al. supplementary material [file S2732494X23004278sup001.docx]

| **Interview Extract** | **Categories** | **Themes & Sub-themes** |
| --- | --- | --- |
| *“There is an antimicrobial stewardship committee that looks into it. It comes under the separate department that is infection prevention and hospital epidemiology. We have a very strong system in place, which looks into it. The stewardship program is head by the chair of the infection control committee who is an adult infectious disease consultant. Within that, the microbiologist is also involved in it. These two guides the program themselves.”* ***(P01- H1)***  *“There is an antimicrobial stewardship committee and there are specific TORs (Terms of references) for that committee duly approved. The committee is chaired by Head Infectious Disease Department. There is the secretary to that committee and the other members include, physicians, microbiologists, there is a position for ID pharmacist vacant, we are in the process of hiring a new one. If you look at the membership, there is a faculty from the IT department, section head representative from the microbiology, pharmacy and infection control department.”****(P01- H2)*** | 1.1.1- Designated Committee | **Theme 1: ASP Structure**  Sub-theme 1.1- ASP Implementer |
| *“We have to be compliant with the stewardship policy. We have certain different components which are defined by the CDC and as well by the IDSA such as one of the intervention is the switch from IV to PO.”* ***(P02– H1)***  *“We do MDRO surveillance. The core responsibility is to provide data related to MDROs that how many cases specific to MDROs were reported. If the cause is related to hospital-acquired infections, we compare and do correlation to the hand hygiene rates because the MDROs spread due to poor hand hygiene. MDROs and hand hygiene data is compared unit wise. Then if there is a unit whose hand hygiene is poor of course, MDROs will increase therefore we work with them for quality improvement.”*  ***(P03- H1)***  *“My goal is to bring down the misuse of antimicrobials. I can only be succeeded with the help of my team of infectious disease trainees and fellows. We conduct rounds twice a week on the patients who are over-treated or under-treated, over-dose or under-dose. The member from the pharmacy joins us; he or she identifies the case where they think the antimicrobials are used appropriately. They provide us with the medical record number, the bed no. of the patient. We then visit these identified patients, try to understand the history and background of the patient. We look at the microbiology whether it was sent appropriately or inappropriately, meaning cultures were contaminated or colonized, was it a real infection or it was a deep tissue culture. We look at the culture and sensitivity report and then come up with the decision.”* ***(P02- H2)*** | 1.1.2- Role and responsibility of stewards |  |
| *“There are infection control nurses and infectious disease physician who are on rotation, they oversee the stewardship activities over weekend and official holidays. On holidays if some restricted or controlled antimicrobials are entered then they do call the ID consultant on the cellular number at home.”* ***(P02- H1)***  *“First of all there is no mechanism for intending medications on the weekend and national holidays. So if any resident get stuck with the treatment then they can call us.”* ***(PO1-H3)*** | 1.1.3- Role and responsibilities of stewards |  |
| *“Yes, we have a policy of ASP at our hospital and we are doing it officially from 2018. The policy is to provide the framework that antimicrobials are used appropriately to reduce resistance.”****(P04– H1)*** | 1.2.1- Availability of formal documented policy/guidelines for antimicrobial stewardship | Sub-theme 1.2: Documentary Support |
| *“The policies are all uploaded on the intranet. Policies are accessible to all the employees. We are very stringent about people knowing how to access these policies so that they can look at it.”* ***(P01 - H1)*** | 1.2.2- Antimicrobial stewardship policy and antimicrobial guidelines available on the intranet |  |
| *“There is a lot of support from the leadership. They are focused on it. It is one of the key quality improvement projects at this point. They do have support from them that’s why the stewardship committee was formed and the whole program was approved by the hospital leadership and we will continue to provide support for it.”* ***(P01- H1)***  *“Initially we did the pilot project at surgical ICU with the help of the hospital leadership. When this pilot project got successful, then there was an official statement by the CEO that we are going to implement the stewardship program in the hospital that from physician X person and pharmacy X person. Then they provided us with the two staff for clinical pharmacist. That’s the support of the hospital leadership for the development and implementation of the program.”* ***(P02 – H1)*** | 1.3.1- Hospital leadership support | Sub-theme 1.3: Leadership Commitment for Antimicrobial Stewardship Program |
| *“Within limits yes we do. By limits, I mean financier budgets. We are committed to it and we do want to continue with the progress. Additional employees are hired specifically to maintain the systems. The leadership has provided 2 man powers that are clinical pharmacists and we are doing some IT interventions. We are in touch with the IT people and four or five interventions would be implemented soon.”* ***(P01 – H1)***  *“There are two or three aspects, number one you talked about IT technology, as we are already a paperless hospital. All the physician orders are computerized and antimicrobial stewardship committee can write their notes and point out why this particular antimicrobial is being stopped or started or why such an amendment is being made. For HI (Health Informative) support is already there. Regarding human resources, whatever the stewardship committee has asked us especially ID pharmacist, the lower staff that would routinely visit the wards, ICUs and critical cares and find out what antimicrobials are being given, and what anomalies are. For all thess resources that they have budgeted, we have already approved. They have our full support in terms of that but the actual success is in the outcome till the time.”* ***(P01- H2)*** | 1.3.2- Hospital leadership commitment for allocation of resources |  |
| *“We review the whole antimicrobial stewardship program. The stewardship committee is one of the major committees at the institute. So the joint staff committee is the apex body for the hospital. So the committee reports to the joint staff committee. That’s the hierarchy come over there.”* ***(P01 – H1)*** | 1.3.3- Periodic follow-up and review of the stewardship program |  |
| *“In core interventions, we have restricted antimicrobials and prospective audits. So there are certain antimicrobials that are restricted, before prescribing them physician has to take the ID approval. There are certain groups of people who can assign or authorize the prescribing of certain antimicrobials including the specialist pharmacist and that are me.”*  ***(P02– H1)*** | 2.1.1- Policy for restricted and controlled antimicrobials | **Theme 2: ASP Intervention**  Core ASP Intervention  Sub-theme 2.1- Formulary Restriction & Pre-authorization |
| *“We do have a documented policy available regarding the restricted and controlled antimicrobials. It is that appropriate cultures and laboratory tests (CBC, Cr etc.) must be sent before starting the antibiotics (restricted or controlled). If the culture results are negative, I.D. consult should be sought in case the primary team wishes to continue the restricted/controlled antibiotic. Moreover, Pharmacy will review orders against usage criteria according to hospital antimicrobial guidelines and if any concern arises, prescribing team will be contacted and concern will be discussed.”* ***(P01 - H1)*** | 2.1.2- *Documented policy for restricted and controlled antimicrobials* |  |
| *“There are certain drug molecules like the restricted ones are fosfomycin IV, tigecycline, linezolid IV/PO, caspofungin, septran IV, antiretroviral medicines (ARV’s) and controlled one is colistin. These drugs need ID approval before the prescription.”* ***(P05- H1)***  *“There are certain drug molecules like we have linezolid, caspofungin, tigecycline, IV fosfomycin, IV septran and anti-retroviral drugs. These drugs need ID approval before the prescription. There are certain combinations like vancomycin with linezolid, vancomycin with cephazolin, two beta-lactam antibiotics and two azole antifungals. So these all require ID approval before prescribing.”* ***(P02 – H1)*** | *2.1.3- -Documented restricted and controlled antimicrobials/ Antimicrobial drugs and regimens require ID approval before prescribing* |  |
| *“The process which we have over here is intending. It takes place every 24 hours so we control irrational prescribing this way. For example, a nurse can only intend in morning hours, as the store is only open until 4:00 pm. For patients, indenting is conducted in morning hours. If the patient is coming in the evening or at night in an emergency, for them we have made a cabinet in our department, which is named as Red Line Trolley, or Cabinet. In that, I have kept little less broad-spectrum antibiotics such as pipracillin- tazobactam, and ceftriaxone, as these are not much restricted antibiotics, this way we restrict the use of antimicrobials at the unit. When I haven’t kept linezolid and colistin then obviously it won’t get prescribed too. So now comes the pre-authorization, in morning if they will ask me that they have to start linezolid so contrary I will ask who has prescribed and who has provided you approval for that. So in this way we conduct pre-authorization.”* ***(P01- H3)***  *“For restricted antimicrobials in life-threatening infections, 24-hour doses can be given. However, afterwards, approval is mandatory and no additional doses will be dispensed from the pharmacy in the absence of authorization. ID physician or ID pharmacist approving any restricted antimicrobial will call the pharmacy to inform regarding approvals, continuation plan or duration of therapy etc. or pharmacist will keep a record of the following information: Patient MR#, name of Doctor providing approval, date and time when the approval is communicated and the pharmacist details who recorded the information in the file.”* ***(P05 - H1)***  “*For controlled antimicrobial, in case of life-threatening infections (with pending cultures), empirical usage (for 72 hours only) is allowed. In this case, pre-authorization from I.D. experts is not required but after 72 hours, for further continuation of therapy, an ID pharmacist or ID physician or medical microbiologist approval is mandatory.”* ***(P01 - H1)*** | 2.1.4- *General prescribing policy for restricted and controlled antimicrobials in life-threatening situation* |  |
| *“Approval system is this whenever the order is entered into the system the pharmacist call the resident that who has approved the drug. Then the resident tells that the doctor “X” has given me the authority of prescription. Otherwise, most of the time whenever ID prescribe these drugs, the ID fellow or ID consultant call themselves in the pharmacy that I have given the approval of this patient with “XYZ” medical record number to me or even I call the pharmacy to inform that this order will get entered for the particular patient so don’t inquire the consultant just process it.”* ***(P02 – H1)***  *“It is only approved by the head of department or any consultant or any faculty member available in the department. As we have, separate wards for male and female and there is a high dependency ward too. So whichever faculty or consultant has a duty at those wards are responsible for the approval but they have to info the stewardship members that is HOD, consultant and clinical pharmacist.”* ***(P01- H3*** | 2.1.5- Approval process for the restricted and control antimicrobials |  |
| *“We carry out prospective audits for certain antibiotics or regimens which are selected by the stewardship subcommittee. These audit regimens are changed on a quarterly basis. We do prospective audits of those patients who are on these regimens. Then we intervene the patients who don’t comply with our guidelines so we do communicate with the consultant as well as with their departmental chief that this patient doesn’t comply with our policy so please either stop or discontinue the antimicrobials or take official ID physician consent.”* ***(P01 - H1)***  *“In general, the broad-spectrum antimicrobials that we have, the audit usually happens in those. This happens with vancomycin, it gets coverage without any use, negative coverage in the broadest spectrum like meropenem and tazobactam type medicines. These combinations are gloomiest things happening mostly during prescription or we can say it is unnecessarily prescribed when not required.”* ***(P02- H2)***  *“Additionally, we keep the check and balance for certain combinations of drugs and should be avoided. Such as two beta-lactam drugs cannot be prescribed together for a synergistic effect. We have even provided education regarding these commonly mistaken synergistic combinations such as two cell-wall inhibitors and same coverage like anaerobes is covered by tazobactam or meropenem then there is no need to prescribe metronidazole and clindamycin until and unless the patient has a liver abscess. The audit for this takes place daily during the round.”* ***(P01- H3)*** |  | Sub-theme 2.2- Prospective Audit and Feedback |
| *“For pharmacy driven interventions we check for culture tests and then switch the patient from IV to PO if patient remains afebrile for 24 to 48 hours with normal TLC count, clinical responses such that patient is not in the shock, patient’s GI is functional and drug bioavailability is 80% to 95%. So these criteria vary from person to person. Moreover, it even saves the cost of healthcare.”* ***(P02– H1)***  *“It happens when we recommend de-escalation or a narrow-spectrum antibiotic. AS team is responsible for this in accordance with the consulting physician. It is based on clinical improvement that is the absence of fever for 48 hours, reduction of white counts perhaps resolution of infiltrates. In addition, we are paying attention to the source of infection while writing the prescription. Especially in surgical services. There may be underlining abscess, narcotizing fasciitis.”* ***(P02- H2)*** | 2.3.1- Parenteral to the oral conversion of antimicrobials | Sub-theme 2.3-Supplemental Elements of Antimicrobial Stewardship Program  Pharmacy Driven Interventions |
| *“These interventions are purely done by the pharmacists. On daily basis, the patients who have renal dysfunctions the lab provide us with the data we do call the consultant and inform regarding it. So they work closely with the different teams at the hospital looking at the drug levels and changes”* ***(P02– H1)*** | 2.3.2- Dose adjustments and dose optimization |  |
| “*Alerts will be issued automatically when the following antibiotics are used in combination since there is significant overlap in the spectrum of these antibiotics. A combination such as Metronidazole IV or Clindamycin with Carbapenems, Piperacillin/tazobactam, Amoxicillin-clavulanate, Cefoperazonesulbactam, Vancomycin IV with Linezolid, Vancomycin IV with Tigecycline, Vancomycin IV with Cloxacillin, Vancomycin IV with Cefazolin, Tigecycline with Linezolid, Voriconazole + Fluconazole, Combination of two beta-lactam antibiotics. If the primary physician chooses to still use these antibiotics together, then an audit may be performed by the ASC to ascertain the appropriateness of this choice. If there are concerns identified by the audit, then a recommendation to either change the regimen or consult ID clinical service will be made. Compliance will be monitored.”* ***(P04 –H1)*** | 2.3.3- Monitoring of overlapping and combination antimicrobials |  |
| *All antibiotics are stopped at five days and need to be re-entered in CPOE that is computerized physician order entry. It is mainly for prophylactic and empiric antibiotics.* ***(P02 – H1)***  *Alerts are generates at the end of the therapy indicating the end of prescribed antimicrobials and in case the physician needs to continue it then they have to inform pharmacy regarding the dose, duration and indication specifically providing the reason to continue.* ***(P03- H2)*** | 2.3.4- Time-sensitive automatic stop orders |  |
| *“We do take educational sessions for all the pharmacists and other healthcare providers (residents) regularly. 3 to 4 sessions in a year is mandatory.”* ***(P01 - H1)***  *“At the departmental level I have conducted antimicrobial stewardship sessions twice for fellows, residents and interns regarding rationalized use of antimicrobials and usage of departmental oriented guidelines. For example, linezolid was used by a lot of doctors very casually over here. It was not realized that it is a very narrow-spectrum antibiotic which one can use it against MRSA or VRE. Now we have educated the doctors enough and I think their numbers have come down. In terms of what couldn’t be used together, it is pretty much written up in our guidelines like IDSA and we read enough about it.”* ***(P02- H2)*** | 2.4.1- Prescriber’s education | Sub-theme 2.4-Education |
| *“We have made a patient education flyer, which is currently with the marketing team. It has been finalized by the stewardship committee and patient education committee. So in this flyer we have mentioned the FAQs regarding antimicrobials like what is antimicrobial, how often do you take and what could be the adverse effects of it. So will be providing education to patients with this flyer.”* ***(P02 – H1)***  *“We conduct counselling for individual patients if they require it. If a patient comes in under our care with the prolong fever and asks us to treat them right way, but, we explain them that we need to make a diagnosis first. It may be an infection, it may be not an infection, and it could be tuberculosis, which is very high up here so we counsel them that we are waiting to establish a diagnosis then we can start treating accordingly. Also when we are taking AS rounds we tell patients that we are just discussing your medication which we think you should be on certain antimicrobials and should be appropriately managed so a little bit of chat with them and we tell them about it.”* ***(P02- H2)*** | 2.4.2- Patient’s education |  |
| *“We have a computerized patient order entry system whenever doctor enters into the system they have to enter the drug name, dose, duration and indication. As far as diagnosis is concerned they do mention it in inpatient progress notes.”* ***(P02– H1)*** | 3.1- Documentation policy for antimicrobial prescriptions | **Theme 3: Medical Record Keeping System** |
| *We have a paperless facility for that matter, we put our notes for sure, there is a pharmacy check, nursing check so yes, and documentation is there. Probably one of the strongest documentation in the city because very few hospitals have any documented things going on. Just because I’ve said that give the medicine so give the medicine, no other documentation takes place at other institutions. It is based the electronic medical health records.* ***(P01- H2)***  *Now what we have changed recently that is the culture reporting methodology. Previously we used to report the positive culture and sensitivities for all the antimicrobials. Now we have curtailed it, on the first screen we just show the panel that we want the physicians to use those antimicrobials only. For example, Carbapenem are not in the first screen, you won’t see any sensitivity pattern to that but there would be penicillin and cephalosporin in it, the common medicines would be there. So that is one change that we have done. Physicians think that these are the common antibiotics. Yes, if there is resistance to that initial panel we move on to the next panel. The third and fourth generation cephalosporin or other advanced molecule antimicrobials will not be reflected at all, that is the change and again for that change.* ***(P01- H2)*** | 3.2- Use of I.T for documentation of antimicrobial prescription |  |
| *“We do monitor it monthly and on regular basis like when they receive antimicrobials when the order is entered, next day me or the other clinical pharmacist who is appointed for this purpose, we do check their login in the pharmacy and the patient progress notes.”* ***(P02 – H1)*** |  |  |
| *“Besides, with the help of an electronic medical record system there is real-time monitoring of the entire prescription takes place whether the entered antimicrobials are properly prescribed in regard to dose, duration and indication. If there are any discrepancies or unusual situation are brought to our attention. Then we deal with it in AS rounds.” (****P02- H2)***  *“We conduct a daily audit for the documentation regarding the prescribed antimicrobial dose, duration and indication during the clinical round. As this unit is 50 bedded, therefore we conduct the audit daily on all admitted patients. We even check regarding whether the prescribed antimicrobial is according to our departmental documented guidelines or not. Moreover, whatever antimicrobials are prescribed at the unit could not be entered without the sign of HOD and a consultant who are the key personals for stewardship activities. Even our departmental nurse informs regarding it as her responsibility is to enter all the antimicrobials in the register when prescribed.”* ***(P01- H3)*** | 3.3- Monitoring of documentation policy |  |
| *“There are certain indicators for the stewardship program. For example restricted antimicrobials, no. of orders, no. of approvals, how many prospective audits took place, no. of intervention done by ASP, the compliance rate of the interventions, supplemental IV to PO interventions: how many patients have been assessed, how much cost saving we have done in terms of IV to PO we have to mention cost-saving. Cost saving from pharmacy is even the indicator for the pharmacy. We do mention regarding cost-saving for IV to PO and dose adjustments that how many patients we have assessed, for how many patients we have asked the consultant to change the dose and how many got accepted. Regarding antimicrobial consumption, the defined daily dose (gms of antimicrobial prescribes and dispensed), we do it quarterly and we do share it with the service lines. Our infection control is doing great work right now. They do monitor the adherence guidelines for handwashing. So we hypothesize that with the implementation of stewardship there is an X drop in the percentage of rates of infections. Right now we have cumulative antibiograms which are issued by the microbiologist every six monthly but right now they are working on departmental antibiogram because departmental sensitivity might be different.”* ***(P02 – H1)*** | 4.1- Quality Measures | **Theme 4: Analyzing and reporting data of antimicrobial stewardship program** |
| *“Periodically the antimicrobial resistance pattern I think every six months is been reported to the hospital staff. In addition, infection control continuously reports infections, infections rates, klebsi rates, pathogens involved in UTI infections. So those rates are reported on a monthly basis and then the trend of these is seen on the annual basis. Moreover, the antibiograms are distributed among prescribers.”* ***(P05- H1)*** | 4.2- Sharing of antimicrobial stewardship indicator reports |  |
| *“We are getting compliance, in restriction, there is 100% compliance, in prospective audits we have got 80-85% compliance, In the initial stage of the stewardship program, this is good compliance. Moreover, for supplemental interventions, we have got above 90%. So this indicates the success story of the stewardship program. For the future, we are thinking about information technology. If we able to restrict our physicians, residents or educate our residents through the IT interventions then it would be the laurel for us.”* ***(P02– H1)*** | 4.3- Impact analysis of antimicrobial stewardship interventions |  |
| **Supplementary Data -** Examples of initial coding, sub-themes and themes | | |
